# α-Synuclein-mediated mitochondrial translocation of cofilin-1 leads to oxidative stress and cell apoptosis in PD

**DOI:** 10.3389/fnins.2024.1420507

**Published:** 2024-08-19

**Authors:** Mingmin Yan, Qian Zhang, Yu Chen, Chenyi Zhu, Dan Wang, Jie Tan, Bihua He, Qin Li, Xiaorong Deng, Yue Wan

**Affiliations:** ^1^Department of Neurology, Hubei No. 3 People's Hospital, School of Medicine, Jianghan University, Wuhan, China; ^2^Hubei Key Laboratory of Cognitive and Affective Disorders, Jianghan University, Wuhan, China

**Keywords:** α-synuclein, cofilin-1, mitochondria, oxidative stress, apoptosis, Parkinson's disease

## Abstract

Parkinson's disease (PD) is characterized by the accumulation of misfolded α-synuclein protein and the loss of dopaminergic neurons in the substantia nigra. Abnormal α-synuclein aggregates form toxic Lewy bodies, ultimately inducing neuronal injury. Mitochondrial dysfunction was reported to be involved in the neurotoxicity of α-synuclein aggregates in PD. However, the specific mechanism by which abnormal α-synuclein aggregates cause mitochondrial disorders remains poorly defined. Previously, we found that cofilin-1, a member of the actin-binding protein, regulates α-synuclein pathogenicity by promoting its aggregation and spreading *in vitro and in vivo*. In this study, we further investigated the effect of cofilin-1 on α-synuclein induced mitochondrial damage. We discovered that α-synuclein aggregates accelerate the translocation of cofilin-1 to mitochondria, promote its combination with the mitochondrial outer membrane receptor Tom 20, and ultimately activate the oxidative damage and apoptosis pathway in mitochondria. All these results demonstrate the important regulatory role of cofilin-1 in the mitochondrial neurotoxicity of pathological α-synuclein during the progression of PD.

## Introduction

Parkinson's disease (PD) is a common neurodegenerative disorder characterized by the degeneration of dopamine neurons in the substantia nigra and the accumulation of Lewy bodies in remaining neurons. Misfolded α-synuclein is a key component of intracellular Lewy bodies in PD. It is widely accepted that the formation of pathological α-synuclein aggregates is characteristic of synucleinopathies and directly induces neuronal damage in PD (Bloem et al., 2021). Dysfunction of multiple organelles, including mitochondria, synaptic vesicles, autophagosomes, lysosomes, is considered to be implicated in the toxic consequences of misfolded α-synuclein aggregates (Wong and Krainc, 2017). However, the specific mechanism induced by pathological α-synuclein remains unclear.

Mitochondrial damage is one of the key pathogenic factors that aggravates the α-synuclein pathology in PD. Mutations in the mitochondrial kinase PINK1 and the mitochondrial-binding protein Parkin were identified as the leading causes of autosomal recessive PD (Giasson and Lee, 2001; Valente et al., 2004; Corti et al., 2011). Moreover, multiple studies *in vitro* showed that the translocation of α-synuclein aggregates to mitochondria coincides with increased mitochondrial dysfunction and neuronal damage (Sulzer, 2001; Choi et al., 2022), revealing important roles of mitochondrial homeostasis in the α-synuclein neurotoxicity of PD.

Cofilin is a member of the actin depolymerizing factor (ADF) family, which mainly binds actin and regulates its cytoskeleton dynamics (Beck et al., 2012; Bravo-Cordero et al., 2013). It is abundant in the central nervous system and widely distributed throughout the cell body and neurites, where it regulates actin dynamics, such as cell division, migration, protein trafficking, and synaptic transmission. The N-terminal serine residue at the third site (Ser-3) in cofilin is considered a conserved phosphorylation site related to the activation of cofilin-1 (Arber et al., 1998; Yang et al., 1998). Cofilin-1 is active when Ser-3 is dephosphorylated and becomes inactive when Ser-3 is phosphorylated. It is worth noting that activated dephosphorylated cofilin is involved in the initiation phase of apoptosis in mitochondria (Klamt et al., 2009). Since cofilin is an essential regulator of cytoskeletal and neuronal functions, disruption of its structure and function has profound implications for several neurological disorders (Shaw and Bamburg, 2017). Cofilin dysregulation in rodent models has long been known to induce dendrite reduction and neurodegeneration, exhibiting many neurological symptoms, including behavioral impairment, memory dysfunction, and sleep deprivation (Bamburg and Wiggan, 2002; Jang et al., 2005; Woo et al., 2012).

In our previous study, cofilin-1 was found to promote the aggregation and transmission of pathological α-synuclein *in vivo and vitro* (Yan et al., 2020, 2022), suggesting cofilin-1 may act as one of the key pathogenic factors that aggravate α-synuclein pathology. However, the roles of cofilin-1 in the pathogenesis of α-synuclein and how cofilin regulates the mitochondrial toxicity of α-synuclein are currently unclear.

In this study, we further investigated the regulatory role of cofilin-1 in α-synuclein neurotoxicity. We discovered that α-synuclein aggregates facilitate the activation and redistribution of cofilin-1 in mitochondria. Meanwhile, cofilin-1 aggravates α-synuclein-induced mitochondrial membrane potential (MMP) disturbance by binding to the mitochondrial outer membrane receptor Tom 20, increasing the generation of reactive oxygen species (ROS) and ultimately inducing the mitochondria-mediated intrinsic apoptosis pathway in cells. Together, these results demonstrate the major role of cofilin-1 in regulating α-synuclein-induced mitochondrial damage.

Pathological α-synuclein can exert its neurotoxicity by inducing the translocation of cofilin-1 to the outer mitochondrial membrane, thereby initiating oxidative stress and cell apoptosis. Our study provides new insights into the mitochondrial neurotoxicity of pathological α-synuclein in PD.

## Methods

### Reagents

Thioflavin T was purchased from Sigma (St. Louis, MO, USA). Lipofectamine-2000, BCA Protein Assay Kit, isopropyl β-D-thiogalactoside (IPTG), and Alexa Fluor 594/488-conjugated secondary antibody were purchased from Thermo Fisher Scientific (Waltham, MA, USA). Mito-track, Caspase 3 Kit, Cell Mitochondria Isolation Kit, rhodamine 123, Hoechst 33342, and propidium iodide (PI) were purchased from Beyotime (Shanghai, China). ROS Kit, MDA Assay Kit, and JC-1 reagents were purchased from Solarbio (Peking, China). Cell Counting Kit-8 (CCK-8) was purchased from Dojindo (Peking, China). In addition, the following reagents and antibodies were used: GAPDH (Proteintech, 60004-1-Ig, Wuhan, China), cofilin (Cell Signaling Technology, 5175, Danvers, MA, USA), p-cofilin (Cell Signaling Technology, 3313, Danvers, MA, USA), S129 (Cell Signaling Technology, 23706, Danvers, MA, USA), α-synuclein (Invitrogen, AHB0261, Carlsbad, CA, USA), ubiqutin (Proteintech, 10201-2-AP, Wuhan, China), Bax (Proteintech, 50599-2-Ig, Wuhan, China), Bcl-2 (Proteintech, 26593-1-AP, Wuhan, China), Cytochrome c (Invitrogen, 45-6100, Carlsbad, CA, USA), Tom 20 (Cell Signaling Technology, 42406, Danvers, MA, USA), HRP-conjugated anti-mouse IgG (BIO-RAD, 170-6516, Hercules, CA, USA), HRP-conjugated anti-rabbit IgG (BIO-RAD, 170-6515, Hercules, CA, USA), Alexa Fluor 594-conjugated goat anti-mouse IgG (Invitrogen, A-11005, Carlsbad, CA, USA), Alexa Fluor 594-conjugated goat anti-rabbit IgG (Invitrogen, A-11012, Carlsbad, CA, USA), DAPI (Biofroxx, EZ3412B205, Germany).

### Protein expression and purification of α-synuclein and cofilin-1

The expression and purification of α-synuclein and cofilin-1 were performed as previously described (Yan et al., 2020, 2022). Briefly, α-synuclein and cofilin-1 coding sequences with 6xHis-tag were cloned into the PRK172 plasmid and transduced into the BL21 *E. coli* strain. Cells were cultured in the Luria-Bertani broth medium with 100 mg/mL ampicillin at 37°C. Then, 0.6 mM isopropyl β-D-thiogalactoside (IPTG) was added until the optical density (OD600) reached approximately 0.8, followed by an additional 6-h incubation for protein expression. Then, the α-synuclein protein and cofilin-1 protein were purified through 6xHis Ni-chelating affinity chromatography and eluted at approximately 125 mM imidazole. The purity level was higher than 90%, as demonstrated through the technique known as sodium dodecyl sulfate-polyacrylamide gel electrophoresis (SDS-PAGE). Protein concentrations were determined by the BCA assay (Thermo Fisher, Waltham, MA, USA). α-Synuclein and cofilin-1 proteins were finally lyophilized and stored at −80°C for further analysis.

### Preparation of fibrils

To prepare fibrils, lyophilized protein was dissolved in phosphate-buffered saline (PBS) and centrifuged at 100,000 rpm for 1 h at 4°C. The residue was removed, and the soluble α-synuclein or cofilin-1 protein in the supernatant was retained. The protein concentration of the supernatant was determined using the bicinchoninic acid (BCA) assay (Thermo Fisher, Waltham, MA, USA). α-Synuclein aggregation was induced by incubating the above-purified α-synuclein protein at 37°C with shaking at 1,000 rpm for 5 days. Mixed fibrils were made of α-synuclein and cofilin-1 proteins and were incubated in a similar manner. α-Synuclein with or without cofilin-1 fibrillization was confirmed using the thioflavin T fluorescence assay. Briefly, aliquots of 5 μl from the incubation samples were diluted to 100 μl with 25 mM thioflavin T in PBS and tested at 450 nm excitation and 510 nm emission using the SpectraMax plate reader. The verified α-synuclein aggregates were then sonicated for 30 s with a 0.5-s pulse on/off (Sonics Vibra cell, Newtown, USA), aliquoted, snap-frozen in liquid nitrogen, and finally stored at −80°C.

### Induction of α-synuclein aggregation (cell seeding experiment)

After transfecting GST-vector and GST-cofilin-1 into 6-well plates with GFP-α-syn-HEK293 cells, 5 μg of α-synuclein fibrils were transduced into cells the next day. For transduction of α-synuclein fibrils, equal volumes of Lipofectamine 2000 (Thermo Fisher, Waltham, MA, USA) and α-synuclein fibrils solutions were diluted in Opti-MEM medium and mixed to a final concentration of 2.8% (v/v), followed by a 20-min incubation before adding into cell medium. Moreover, 48 h later, the formation and morphology of endogenous α-synuclein inclusions were observed using fluorescence microscopy at different times after adding fibrils.

### Primary neuron cultures

Primary mouse cortical neurons dissected from α-synuclein A53T transgenic mice embryos were cultured as previously described. The neurons were infected with adeno-associated viruses (AAVs) or α-synuclein fibrils at 5 days *in vitro*. 1 week later, the neurons were fixed in 4% formaldehyde and stained as below.

### Immunofluorescence for neurons and cells

For immunofluorescence, neurons and cells were first fixed and permeabilized with 4% paraformaldehyde supplemented with 1% TX-100 (v/v) for 15 min. The permeabilization solution was then removed and washed three times with PBS. After blocking with a 3% BSA solution at room temperature for 30 min, primary antibodies were added and incubated overnight at 4°C. Coverslips were rinsed three times in PBS for 15 min. Subsequently, the samples were incubated using Alexa Fluor 488/594 anti-mouse/rabbit secondary antibody (1:500) for 1 h and washed three times with PBS. Then, the slides were stained with DAPI (300 nM) for 30 s, washed with PBS again, and finally mounted on glass slides. These slides were observed under an Olympus inverted fluorescence microscope (Olympus TH4-200, Japan). The experiment was repeated at least three times.

### Immunostaining for brain samples

The paraffin slices of mouse brain samples were treated with 0.3% H_2_O_2_ for 10 min. Then, the slices were washed three times in PBS, blocked in 3% BSA for 1 h, and then incubated overnight with primary antibodies at 4°C. The signals were developed with the Histostain-SP kit (Invitrogen, Carlsbad, CA, USA). To detect the colocalization of cofilin-1 and Tom 20 in α-synuclein A53T transgenic mice, the sections were incubated with corresponding primary antibodies overnight at 4°C. Then, the sections were washed three times in PBS and incubated with Alexa Fluor 488/594 anti-mouse/rabbit fluorescent secondary antibody (1:500) for 1 h at room temperature. After being stained with DAPI, the sections were covered with a glass cover using the mounting solution and examined under a fluorescence microscope.

### Cell viability assay

Cell viability was detected using the Cell Counting Kit-8 viability assay. Following the manufacturer's instructions, the cells were first transduced with α-synuclein fibrils and mixed fibrils, and then plated at a density of 1 × 10^4^ cells/100 μL in 96-well plates. After 48 h, 10 μL of CCK8 solution was added to each well and incubated at 37°C for 2 h. The medium was then removed and washed twice with PBS. The absorbance was detected at 450 nm by using a Spectra Max Plus 384 Microplate Reader. Cell viability was expressed as the percentage of the control group. The experiment was repeated at least three times.

### Western blot analysis

Cells were washed twice with PBS and scraped into an ice-cold NP40 cell lysis buffer containing protease and phosphatase inhibitors for 30 min. The lysate was centrifuged at 15,000 rpm for 15 min at 4°C. The protein concentration was determined in the BCA assay. The supernatant was boiled in an SDS loading buffer. The SDS protein loading buffer (6X) contained 375mM Tris-HCl with pH 6.8, 9% SDS, 50% glycerin, and 0.03% bromophenol blue, and it was diluted to 25 ml with ultrapure water. The samples were separated on SDS/15% polyacrylamide gels, transferred to nitrocellulose membranes (Thermo Fisher Scientific, Waltham, MA, USA) using a semi-dry system (Bio-Rad, Hercules, CA, USA), blocked in 5% non-fat milk (50 mg/ml) in Tris Buffered Saline with Tween-20 (TBST), and incubated with primary antibodies overnight at 4°C. The membranes were washed three times with TBST and then labeled with horseradish peroxidase (HRP)-conjugated secondary antibodies. The signals were detected using enhanced chemiluminescent (ECL) substrates. Image J software was used to measure the density of Western blot bands. The experiment was repeated at least three times.

### Triton-X and SDS fractionation of soluble and insoluble α-synuclein

GST-vector and GST-cofilin-1 were transfected into HEK293 cells separately. α-Synuclein fibrils were transduced into cells on the second day. Moreover, 48 h later, the cells were collected. For sequential extraction of soluble and insoluble α-synuclein, the cells were washed twice with PBS and scraped into 1% Triton X-100 (TX-100) containing protease and phosphatase inhibitors. After sonication using a fine probe [0.5-s pulse at an amplitude of 20%, repeated 10 times (using a Ningbo Toshiba Ultrasonic Cell Crusher JY99-IIDN, China)], cell lysates were incubated on ice for 30 min and centrifuged at 100,000 g for 30 min at 4°C. The supernatant (TX-100 soluble fraction) was collected while the pellet was washed in 1% Triton X-100, sonicated as described earlier, and then centrifuged for another 30 min at 100,000 g. The supernatant was discarded, whereas the pellet (TX-100 insoluble fraction) was resuspended in 2% sodium dodecyl sulfate (SDS) supplemented with protease and phosphatase inhibitors. It was sonicated using a fine probe (0.5-s pulse at an amplitude of 20%, 15 times). Then, the lysate was lysed at room temperature for 30 min and centrifuged. The supernatant was retained as the insoluble fraction. The experiment was repeated at least three times.

### Mitochondrial and cytoplasmic extraction

Differential centrifugation was employed to extract mitochondria and cytoplasm from HEK293 cells using the Beyotime Cell Mitochondria Isolation Kit to obtain the cytoplasmic and mitochondrial fractions. According to the manufacturer's instructions, HEK293 cells were lysed in a precooled mitochondrial separation reagent and centrifuged at 600 g for 5 min at 4°C. Subsequently. The supernatant was centrifuged at 11,000 g for 10 min at 4°C to isolate cytoplasmic and mitochondrial fractions. Then, the cytoplasmic supernatant and the pellet containing isolated mitochondria were carefully collected. The mitochondrial preparation was washed several times and resuspended in a mitochondrial stock solution. Finally, the mitochondrial lysate supplemented with the protease inhibitor was prepared for protein analysis.

### Determination of intracellular ROS and MDA

To determine the intracellular ROS level, SH-SY5Y cells were incubated with 50 μmol/L DCFH-DA (2′,7′-dichlorodihydrofluorescein diacetate) at 28°C for 30 min in the dark and then washed twice with the PBS buffer. Fluorescent images of intracellular ROS were captured using a fluorescence microscope. According to the manufacturer's instructions, intracellular MDA levels were determined using the Malondialdehyde (MDA) assay kits. First, the treated cells were resuspended in distilled deionized water and the thiobarbituric acid reagent and then heated for 15 min in a boiling water bath. The mixtures were then cooled to room temperature and centrifuged at 3,000 rpm for 5 min to remove the cell debris. The absorbance of the supernatants was measured at 535 nm, and non-specific turbidity was corrected by subtracting the absorbance at 600 and 450 nm.

### Detection of mitochondrial membrane potential

Changes in the mitochondrial membrane potential were measured using rhodamine and JC-1 staining methods. Rhodamine 123 staining working solution was added into the SY5Y cells and then incubated at 37°C in a cell culture incubator for 40 min. After being washed twice with cell culture medium, the SY5Y cells were observed under a fluorescence microscope for mitochondrial membrane potential analysis. In addition, a Solarbio mitochondrial membrane sensor kit was used to determine the mitochondrial membrane potential by measuring the potential-dependent accumulation of 5,5′,6,6′-tetrachloro-1,1′,3,3′-tetraethylbenzimidazolylcarbocyanine iodide (JC-1). JC-1 emits red fluorescence while aggregated in the mitochondria of healthy cells. Nevertheless, the dye cannot accumulate in the mitochondria of cells with a collapsed MMP and remains as a monomer, emitting green fluorescence throughout the cell. After being treated with α-synuclein fibrils, cofilin-1, or cofilin mutants for 36 h and washed with PBS, the cells were cultured with JC-1 solution for 20 min at 37°C. Then, the fluorescence intensity was determined using fluorescence microscopy. These experiments were repeated at least three times.

### Statistical analyses

All data were expressed as mean ± SEM (standard error of the mean). The statistical analysis was performed using Student's *t*-test for a two-group comparison. When variances were not equal, alternative statistical methods, such as the Mann–Whitney test, were applied. A one-way ANOVA was applied to confirm the significant effects among three or more groups, followed by Tukey's multiple comparisons for *post hoc* tests. A two-way ANOVA was used to analyze the significant effects of grouped data, followed by Bonferroni's multiple comparisons for *post hoc* tests. For the data that were not to be normally distributed, the Kruskal–Wallis test was employed for comparisons, and GraphPad Prism software was used in the above statistical analyses. Differences with *P* < 0.05 were considered significant. All experiments were performed in triplicate for at least three independent trials.

## Results

### Cofilin-1 promotes the aggregation of α-synuclein *in vitro* and in cells

**Figure 1:**
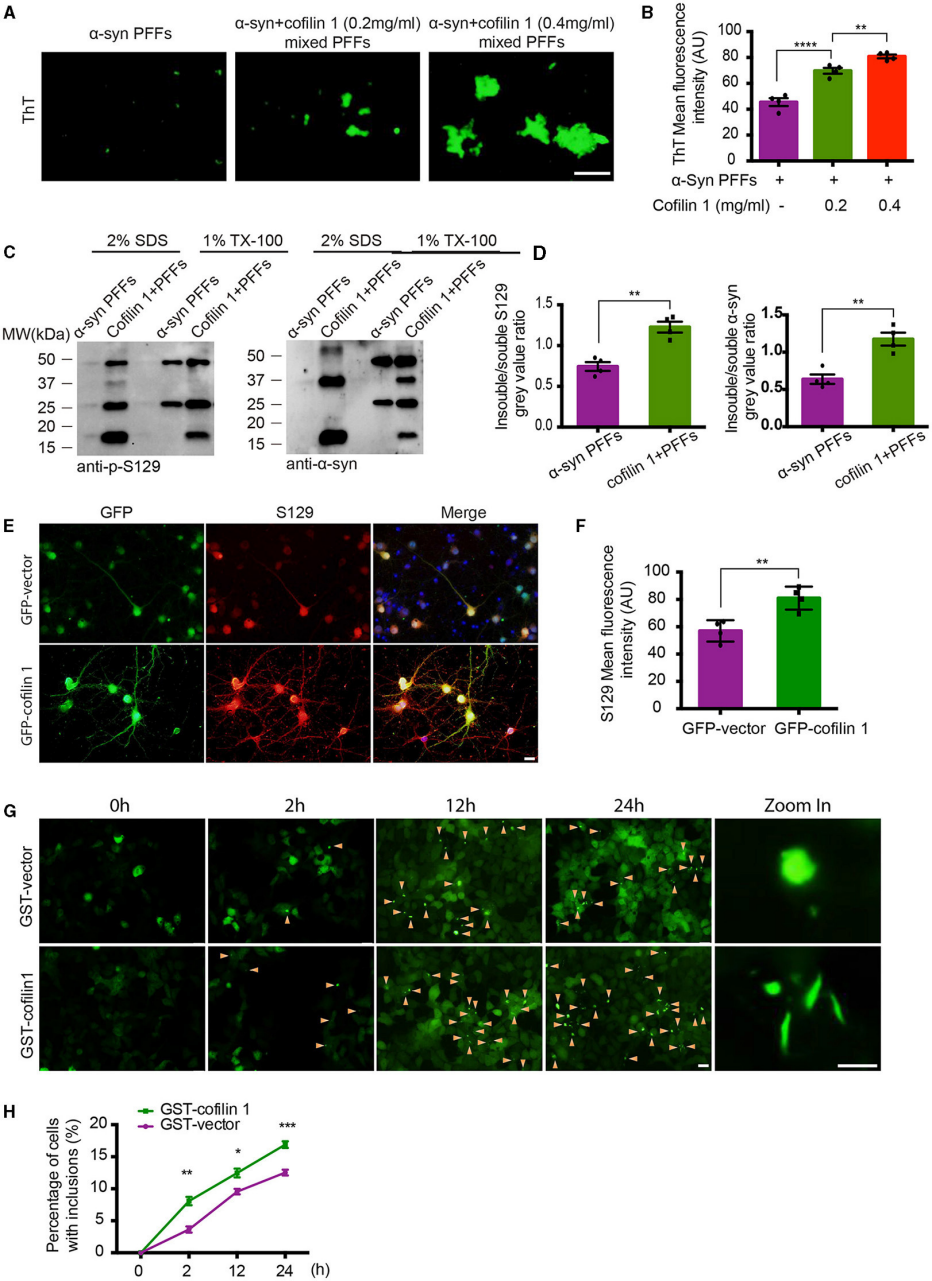
Cofilin-1 promotes the aggregation of α-synuclein *in vitro* and in cells. **(A, B)** Thioflavin T fluorescent staining showing α-synuclein aggregation with cofilin-1 in different concentrations. Mixed pre-formed fibrils (PFFs) composed of α-synuclein and cofilin-1 were more aggregated than pure α-synuclein PFFs, especially at high concentrations of cofilin-1. Scale bar, 100 μm. Data are represented as mean ± SEM, ^**^P < 0.01, ^****^P < 0.0001 by one-way ANOVA. **(C, D)** Western blot analysis of soluble and insoluble p-S129 α-synuclein using triton-X and SDS dissolution test. Compared with pure α-synuclein PFFs, overexpressed cofilin-1 and α-synuclein PFFs in HEK293 cells induced more insoluble α-synuclein aggregates. Quantification of insoluble S129 and α-synuclein. The results are normalized to soluble S129 and α-synuclein. Data are represented as mean ± SEM, ^**^P < 0.01 by *t*-test. **(E, F)** Immunostaining for S129 shows that overexpressed cofilin-1 promoted the phosphorylation of α-synuclein in neurons of α-synuclein A53T mice. Scale bar, 20 μm. Data are represented as mean ± SEM, ^**^P < 0.01 by *t*-test. **(G)** Cell seeding experiment for intracellular α-synuclein aggregates. Green aggregates (arrows) indicate α-synuclein aggregates. The aggregates in the overexpressed cofilin-1 group were significantly increased at different times compared with the control group, while the aggregate morphology was also inconsistent. Scale bar, 20 μm; Zoom in Scale bar, 5 μm. **(H)** Counting of cells with α-synuclein inclusions. At least 120 cells from at least three fields were counted in the experiments. Data are represented as mean ± SEM, ^*^P < 0.05, ^**^P < 0.01, and ^***^P < 0.001 by two-way ANOVA.

In our previous study, since purified cofilin-1 *in vitro* was considered to promote the aggregation kinetics of α-synuclein, we further observed the morphology of α-synuclein aggregates with or without cofilin-1 using thioflavin T (ThT) fluorescence staining. The pathological α-synuclein was found to accumulate more abundantly as the concentration of cofilin-1 increased (Figures 1A, B). Furthermore, compared with the pure α-synuclein fibrils, overexpressed cofilin-1 and α-synuclein fibrils in human embryonic kidney 293 (HEK293) cells induced more expression of higher molecular weight species, especially in the insoluble 2% SDS solution (Figures 1C, D). This result represented the overexpression of cofilin-1 together with α-synuclein fibrils, which promoted added aggregation of endogenous α-synuclein in HEK293 cells. Immunostaining of p-s129 in neurons of α-synuclein A53T mice also supported this result; adeno-associated virus encoding EGFP-cofilin-1 (AAV-GFP-cofilin-1) markedly strengthened the immunoreactivity of p-S129 in neurons compared with the control AAVs (AAV-GFP-vector) (Figures 1E, F). To further investigate the regulation of cofilin-1 on the aggregation of α-synuclein in cells, we used the HEK293 cell line stably transfected with YFP-α-synuclein A53T (YFP-α-syn-HEK293 cells) as reporter cells for the cell seeding experiment. While these cells are transduced with exogenous α-synuclein fibrils, endogenous α-synuclein aggregates into inclusions with visible green fluorescence. We transfected GST-vector and GST-cofilin-1 into the reporter cells, respectively, and transduced with exogenous α-synuclein fibrils the next day. Interestingly, we found that the cells developed globular inclusions only 2 h after treatment with α-synuclein fibrils. The number of intracellular α-synuclein aggregates increased gradually over time, especially when cofilin-1 was overexpressed. However, the overexpression of cofilin-1 promoted the aggregation of endogenous α-synuclein to form different rod-shaped inclusions rather than the globular shapes (Figures 1G, H), suggesting that cofilin-1 promotes the aggregation of α-synuclein into more distinct inclusions, not only *in vitro* but also in cells.

### α-Synuclein fibrils facilitate the activation and redistribution of cofilin-1

**Figure 2:**
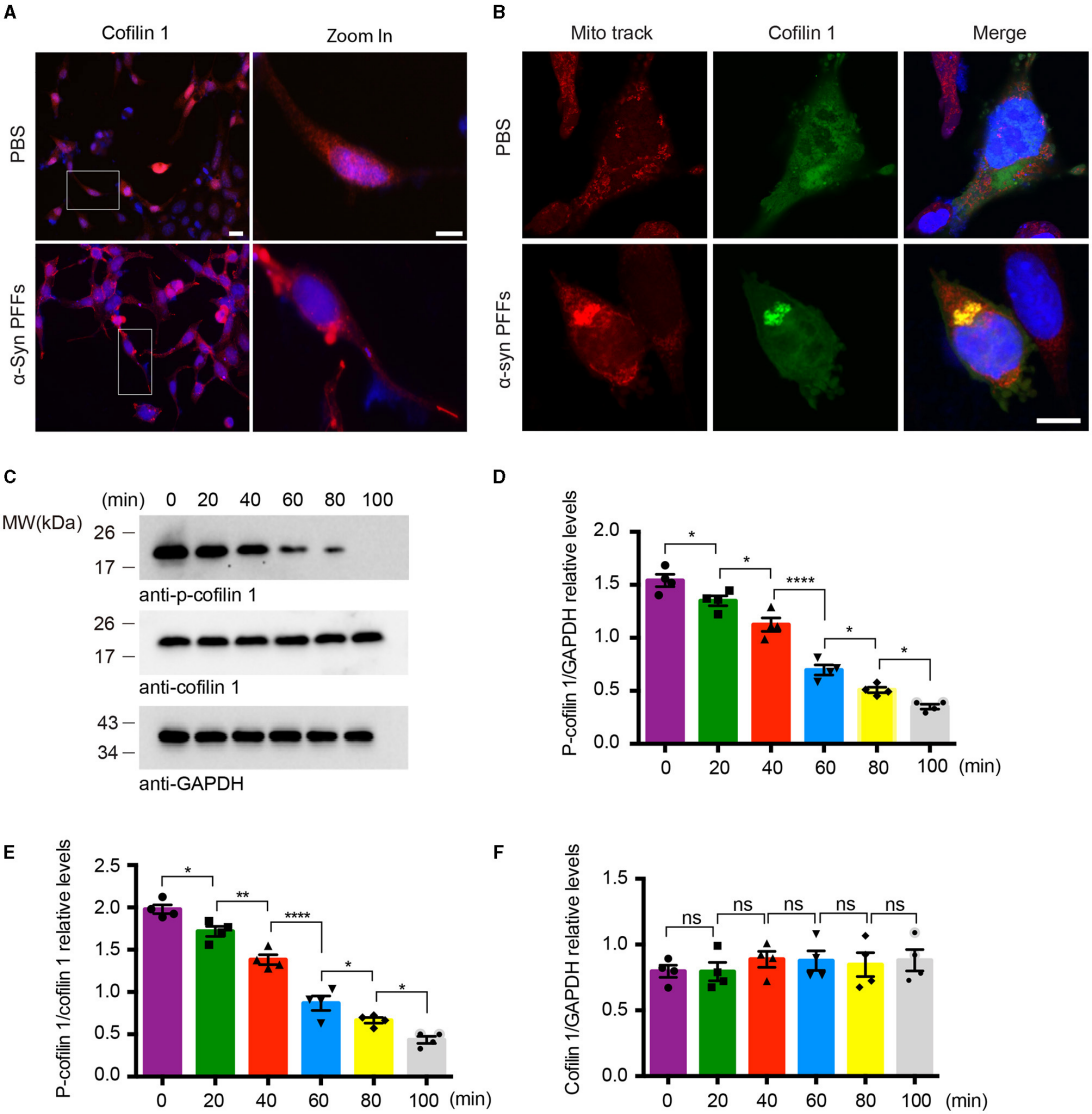
α-Synuclein fibrils facilitate the activation and redistribution of cofilin-1. **(A)** Immunostaining showing the redistribution of cofilin-1 in YFP-α-syn-HEK293 cells with α-synuclein fibrils. Scale bar, 20 μm; Zoom in Scale bar, 10 μm. **(B)** Immunofluorescence showing the colocalization of cofilin-1 and Mito-track in SH-SY5Y cells transduced with α-synuclein fibrils. Scale bar, 10 μm. **(C–F)** Western blot detection of p-cofilin-1, total cofilin-1, and GAPDH in HEK293 cells. The expression of non-activated p-cofilin-1 decreased at different times after transducing α-synuclein fibrils. Data are represented as mean ± SEM, *P < 0.05, **P < 0.01, ****P < 0.0001. ns, not statistically significant by one-way ANOVA.

Since cofilin-1 promotes the aggregation of α-synuclein, we further explored whether α-synuclein aggregates cause their neurotoxicity through cofilin-1. We examined the expression of cofilin-1 in YFP-α-syn-HEK293 cells with α-synuclein fibrils and found that cofilin-1 was mainly expressed in the cytoplasm and protrusions rather than evenly distributed in cells (Figure 2A), suggesting that α-synuclein fibrils promote the redistribution of endogenous cofilin-1. We further verified the effect of α-synuclein fibrils on cofilin-1 in human neuroblastoma cells (SH-SY5Y cells). After transduction of α-synuclein fibrils, we found that cofilin-1 colocalized with the mitochondrial fluorescent probe (Mito-track) (Figure 2B), suggesting that α-synuclein fibrils promote the expression of cofilin-1 in mitochondria. Finally, we transduced HEK293 cells with α-synuclein fibrils and collected cells at different times after adding fibrils. The Western blot analysis showed that the level of p-cofilin-1 gradually declined, while the expression of total cofilin-1 was almost the same (Figures 2C–F). This observation suggests that α-synuclein fibrils induce the decrease in inactive p-cofilin, resulting in an increase in activated cofilin-1. Overall, our results indicate that α-synuclein fibrils facilitate the activation and redistribution of cofilin-1 in cells.

### Cofilin-1 aggravates α-synuclein-induced oxidative stress in cells

**Figure 3:**
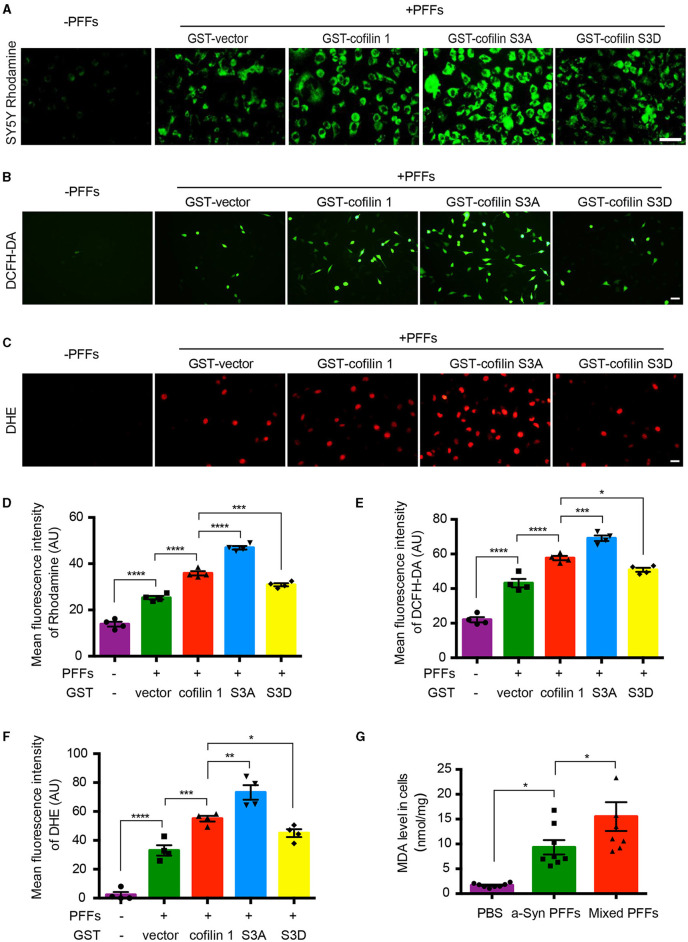
Cofilin-1 aggravates α-synuclein-induced oxidative stress in cells. **(A, D)** Detection of the mitochondrial membrane potential in SH-SY5Y cells with Rhodamine. Continuously activated cofilin S3A, but not phosphorylated cofilin S3D, triggered more mitochondrial membrane damage in SH-SY5Y cells. Scale bar, 50 μm. Data are represented as mean ± SEM, ***P < 0.001, ****P < 0.0001 by one-way ANOVA. **(B, E)** Intracellular ROS was detected by the DCFH-DA probe in SH-SY5Y cells. Cofilin S3A triggered more DCFH (green fluorescence), while cofilin S3D weakened its generation. Scale bar, 50 μm. Data are represented as mean ± SEM, *P < 0.05, ***P < 0.001, ****P < 0.0001 by one-way ANOVA. **(C, F)** Intracellular ROS was detected by the DHE probe in SH-SY5Y cells. DHE (red fluorescence) was reduced in the cofilin S3D group, while cofilin S3A induced more DHE fluorescence intensity. Scale bar, 20 μm. Data are represented as mean ± SEM, *P < 0.05, **P < 0.01, ***P < 0.001, and ****P < 0.0001 by one-way ANOVA. **(G)** Quantification of MDA after fibrils transduced into SH-SY5Y cells. The mean MDA values of the PBS, α-synuclein fibrils, and mixed fibrils groups were 1.677, 9.33, and 15.51, respectively. Data are mean ± SEM, *P < 0.05 by one-way ANOVA.

It has been reported that cofilin-1 initiates the apoptotic pathway and induces oxidative stress in mitochondria. We further verified whether α-synuclein exerts its toxic effects through cofilin-1. We first constructed the point mutation of cofilin-1 as cofilin S3A and cofilin S3D. Cofilin S3A is a continuously activated state, whereas cofilin S3D cannot be activated. Then, we examined the mitochondrial membrane potential in SH-SY5Y cells with Rhodamine. After transducing SH-SY5Y cells with pure α-synuclein fibrils, together with cofilin-1, cofilin S3A, or cofilin S3D separately, the fluorescence intensities were generally increased in α-synuclein fibrils compared with the control group, indicating that the fibrils triggered the mitochondrial membrane damage. Moreover, the overexpression of cofilin-1 exacerbates the loss of mitochondrial membrane potential, especially when dephosphorylated cofilin S3A was overexpressed, while phosphorylated inactive cofilin S3D decreased the loss of membrane potential in mitochondria (Figures 3A, D). Moreover, we stained SH-SY5Y cells using the ROS fluorescence probe DCFH-DA (green) and DHE (red). The fluorescence levels of DCFH-DA and DHE were increased in fibril groups compared with the control group. Similarly, α-synuclein fibrils with the overexpression of cofilin triggered stronger fluorescence intensity than pure α-synuclein fibrils, especially fibrils with cofilin S3A, but the fluorescence intensity was decreased in α-synuclein fibrils with the cofilin S3D group, indicating that ROS levels increased when cofilin-1 was overexpressed with α-synuclein fibrils. Dephosphorylated active cofilin-1 accelerates more severe ROS generation induced by α-synuclein fibrils, whereas inactive cofilin-1 reduces this α-synuclein-induced oxidative stress damage in cells (Figures 3B, C, E, F). Consistent with these results, the MDA level in α-synuclein fibrils group increased more significantly than the control group, while mixed fibrils of α-synuclein and cofilin-1 attenuated the MDA level in SH-SY5Y cells (Figure 3G). These findings suggest that cofilin-1 aggravates oxidative stress induced by α-synuclein fibrils in cells, especially active cofilin-1 instead of phosphorylated inactive cofilin-1.

### Overexpression of cofilin-1 accelerates α-synuclein-induced cell apoptosis

**Figure 4:**
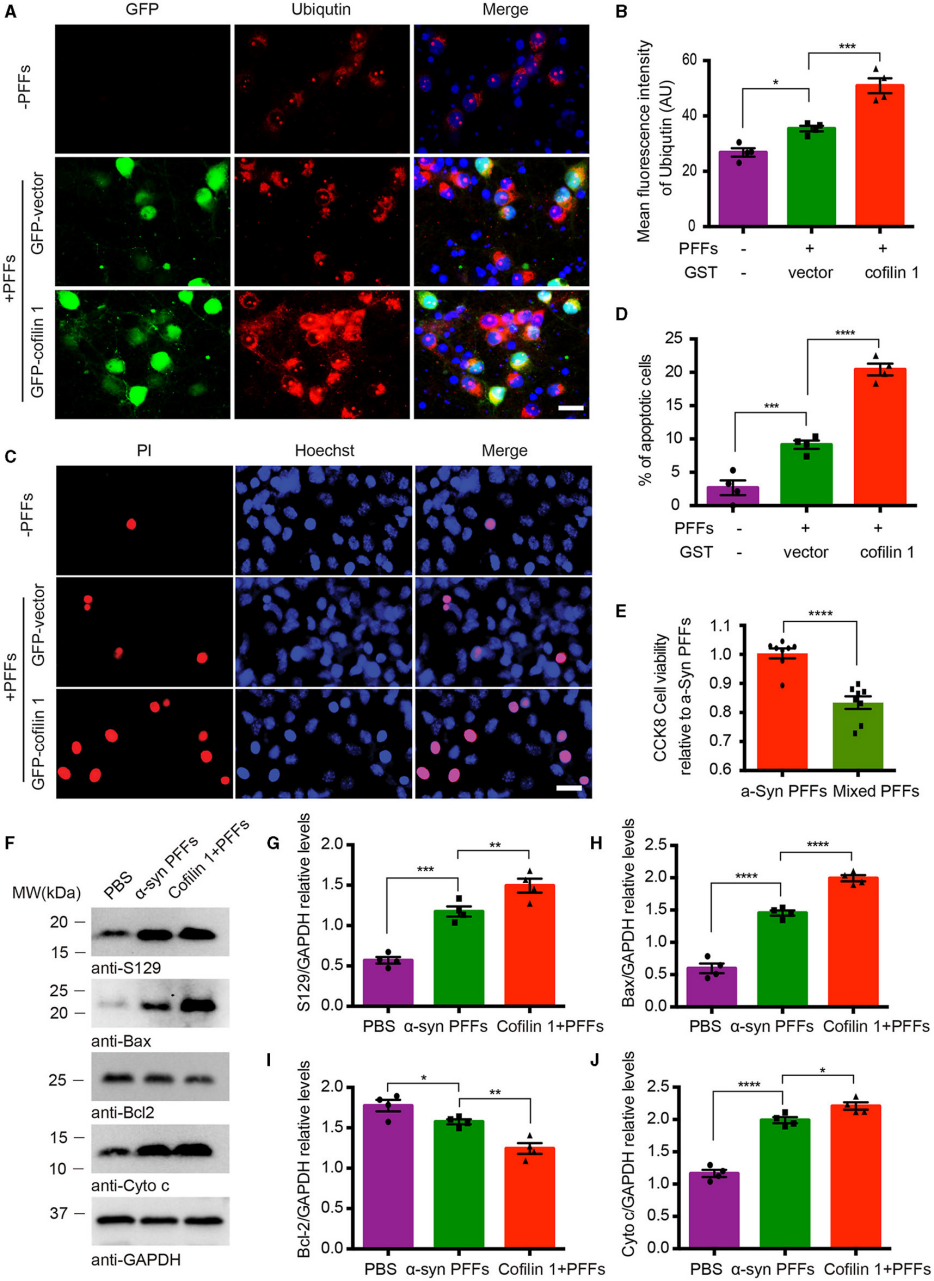
Overexpression of cofilin-1 accelerates α-synuclein-induced cell apoptosis. **(A, B)** Immunostaining showing the expression of ubiquitin in neurons of α-synuclein A53T transgenic mice. Overexpressed cofilin-1 promoted the expression of ubiquitin. Scale bar, 20 μm. Data are represented as mean ± SEM, *P < 0.05, ***P < 0.001 by one-way ANOVA. **(C, D)** Detection of cell apoptosis in SH-SY5Y cells via Hoechst staining. Overexpression of cofilin-1 accelerated α-synuclein-induced cell apoptosis. Scale bar, 20 μm. Data are represented as mean ± SEM, ***P < 0.001, ****P < 0.0001 by one-way ANOVA. **(E)** CCK8 analysis shows decreased cell viability with mixed fibrils in SH-SY5Y cells. Mixed fibrils reduced the cell viability compared to pure α-synuclein fibrils. Data are represented as mean ± SEM, ****P < 0.0001 by *t*-test. **(F–J)** Western blot analysis of p-S129 and apoptosis-related proteins Bax, Bcl-2, and cytochrome c. Cytochrome c was tested in the cytosolic fraction, while Bax and Bcl-2 were detected in mitochondria. Quantitative analysis is normalized to GAPDH. Data are represented as mean ± SEM, *P < 0.05, **P < 0.01, ***P < 0.001, and ****P < 0.0001 by one-way ANOVA.

We further investigated the effect of cofilin-1 on cell apoptosis induced by α-synuclein fibrils. After the neurons of α-synuclein A53T transgenic mice treated with AAV-GFP-cofilin-1 and AAV-GFP-vector, we found that the reactivities of proteolysis marker ubiquitin were much stronger in neurons with AAV-GFP-cofilin-1 than that in neurons with control AAVs, suggesting that overexpressed cofilin-1 promotes the degradation of proteins (Figures 4A, B). We also assessed the effect of cofilin-1 on apoptosis in SH-SY5Y cells via Hoechst staining. We found that the α-synuclein fibrils group induced more cell apoptosis than the control group, while the apoptotic rate in cells transduced with GFP-cofilin-1 was higher than that with GFP-vector (Figures 4C, D). Similarly, compared with the pure α-synuclein fibrils, the cell viability of mixed fibrils declined significantly, indicating that cofilin-1 reduces cell viability and accelerates α-synuclein-induced cell apoptosis (Figure 4E). To further verify the effect of cofilin-1 on toxic α-synuclein fibrils, we detected the expression of p-S129 and apoptosis-related proteins, Bax, Bcl-2, and cytochrome c. It was well known that proapoptotic protein Bax, anti-apoptotic protein Bcl-2, and cytochrome c played an important role in cell apoptosis. Bcl-2 and Bax proteins were considered to regulate the release of cytochrome c from mitochondria into the cytoplasm, which ultimately triggered the executive apoptotic pathways. As expected, α-synuclein fibrils with transfected cofilin-1 induced more expression of p-S129, Bax, and cytochrome c, while mitochondrial Bcl-2 was significantly reduced in the presence of cofilin-1 (Figures 4F–J). These results suggest that cofilin-1 accelerates α-synuclein-induced cell apoptosis and neurotoxicity.

### Cofilin-1 binds tom 20 to promote mitochondrial damage and cell apoptosis

**Figure 5:**
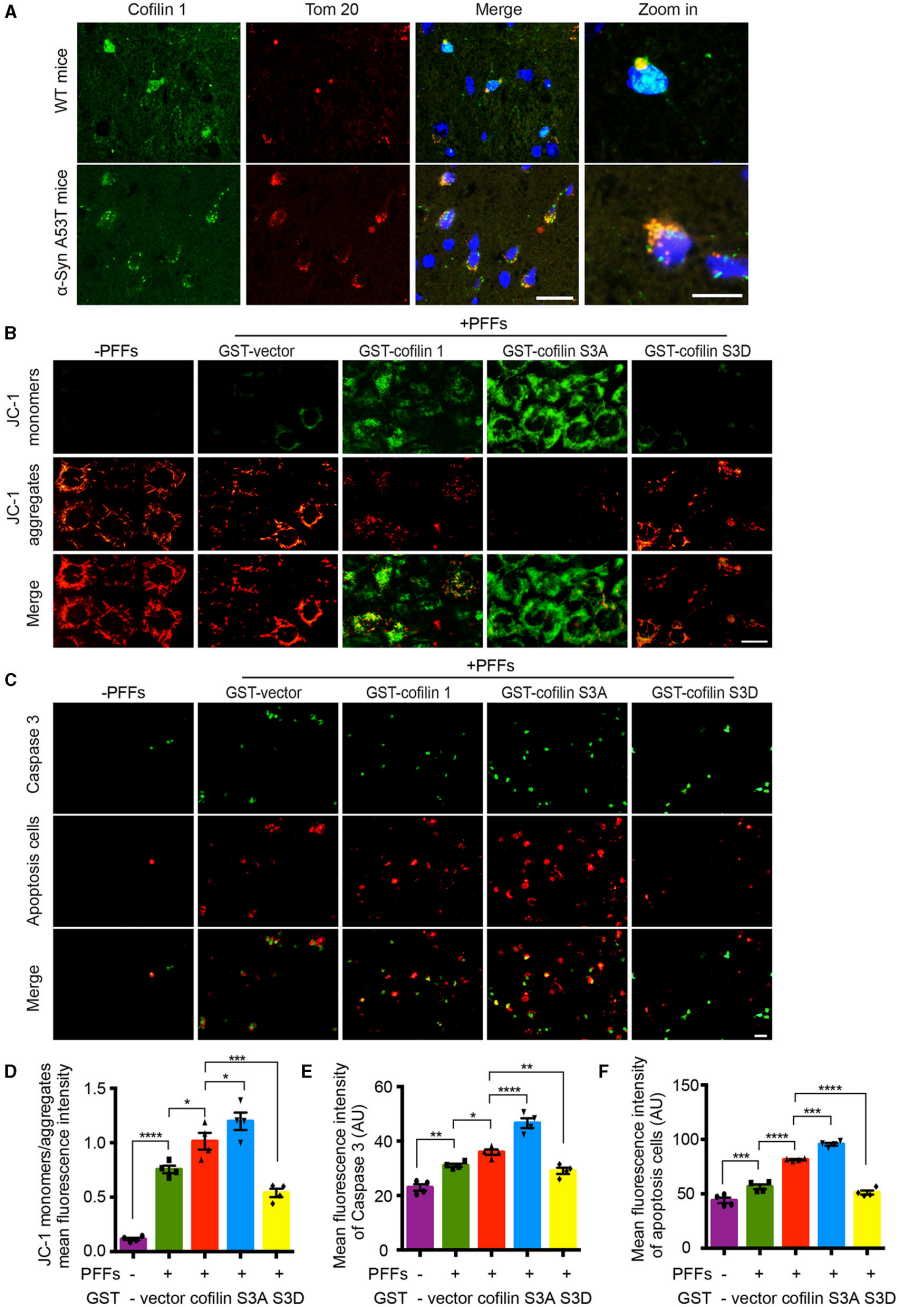
Cofilin-1 binds Tom 20 to promote mitochondrial damage and cell apoptosis. **(A)** Immunofluorescence showing the colocalization of cofilin-1 and Tom 20 in α-synuclein A53T transgenic mice. Scale bar, 20 μm; Zoom in Scale bar, 5 μm. **(B, D)** JC-1 kits detect the mitochondrial membrane potential in HEK293 cells. Active cofilin S3A aroused more JC-1 monomers, while inactive cofilin S3D attenuated the expression of JC-1 monomers. Quantification of JC-1 monomers/JC-1 aggregates. Scale bar, 20 μm. Data are represented as mean ± SEM, *P < 0.05, ***P < 0.001, and ****P < 0.0001 by one-way ANOVA. **(C, E, F)** Caspase 3 staining detecting apoptosis in HEK293 cells. Cofilin-1, especially active cofilin S3A, induced more expression of caspase 3 and cell apoptosis. Scale bar, 50 μm. Data are represented as mean ± SEM, *P < 0.05, **P < 0.01, ***P < 0.001, and ****P < 0.0001 by one-way ANOVA.

Since cofilin-1 accelerates the oxidative stress and cell apoptosis induced by α-synuclein fibrils, we further tested how cofilin-1 regulates its toxicity. We detected the expression of cofilin-1 in α-synuclein A53T transgenic mice. Immunofluorescence staining revealed that cofilin-1 was colocalized with Tom 20 in the brains of α-synuclein A53T mice, whereas cofilin-1 was scattered in the cytoplasm of wild-type mice (Figure 5A), suggesting that α-synuclein promotes the location of cofilin-1 to mitochondria and binds Tom 20. It is consistent with our previous results. Moreover, we further detected the mitochondrial membrane potential damage and apoptosis using the JC-1 assay and caspase-3 kits respectively. The decrease in mitochondrial membrane potential is an early marker event of cell apoptosis. Decreased JC-1 aggregates and increased JC-1 monomers suggest mitochondrial membrane potential damage. We found that α-synuclein fibrils alone induced few immunofluorescences of JC-1 monomers and caspase 3, while cofilin-1, especially active cofilin S3A, aroused much stronger positive immunostaining. Inactive cofilin S3D attenuated the expression of JC-1 monomers and caspase 3 in cells (Figures 5B–F). All these results suggest that cofilin-1 may combine with Tom 20 and induce loss of mitochondrial membrane potential and finally lead to cell apoptosis.

## Discussion

PD is an age-related neurodegenerative disorder characterized by the presence of misfolded α-synuclein in neurons and dopaminergic neuron loss in the substantia nigra. Although α-synuclein normally localizes to the presynaptic terminal, its toxic aggregates localize throughout the cell body, which suggests that multiple organelles might be implicated in the α-synuclein toxicity (Wong and Krainc, 2017). Mitochondria are crucial for adenosine triphosphate (ATP) synthesis, calcium storage, lipid metabolism, and neuronal survival (Galluzzi et al., 2012). Mitochondrial dysfunction is the primary step leading to neuronal injury in PD (Exner et al., 2012; Ryan et al., 2015). Numerous reports have described mitochondrial dysfunction in models of pathological α-synuclein (Pech and Verstreken, 2018). α-Synuclein toxicity might directly disrupt mitochondrial homeostasis, given that mice with A53T α-synuclein mutations have increased mitochondrial damage, while dopaminergic neuron degeneration is prevented in mice lacking α-synuclein (Wong and Krainc, 2017). However, there is currently no consensus on how α-synuclein causes its mitochondrial damage.

Cofilin-1 is considered to be activated via dephosphorylation at Ser-3 by slingshot proteins (SSHs) and gets inactivated via phosphorylation by LIM kinases (LIMKs). Although the mechanism of cofilin-mediated actin dynamics has been known for decades, recent studies suggest the profound impacts of cofilin-1 in neurodegenerative diseases. In our previous studies, cofilin-1 was found to be involved in the aggregation and transmission of α-synuclein *in vitro and vivo* (Yan et al., 2020, 2022). However, whether cofilin-1 is involved in the mitochondrial damage of α-synuclein is unknown. Therefore, we investigated the role of cofilin-1 in α-synuclein-mediated mitochondrial impairment in this study. We gained insights into the role of cofilin in α-synuclein-induced mitochondrial dysfunction in PD. We made several novel observations demonstrating that α-synuclein aggregates regulate the distribution of cofilin-1 to mitochondria and cofilin-1 is critical for α-synuclein-induced mitochondrial damage and cell apoptosis. Our findings highlight the critical importance of the cofilin pathway in PD pathogenesis.

Moreover, it has been reported that the translocation of cofilin to mitochondria requires its activated dephosphorylation state, and activated cofilin is necessary for the release of cytochrome c and Bax translocation in mitochondria, which represents the initiation of apoptosis (Chua et al., 2003; Posadas et al., 2012). Multiple studies have confirmed the important role of cofilin-1 in the regulation of apoptosis in mitochondria, where it contributes to mitochondrial membrane depolarization, permeabilization, and the release of proapoptotic factors, thereby leading to neuronal death (Liu et al., 2017). Knockdown of either cofilin or Slingshot phosphatase has a marked neuroprotective effect on neuronal death (Madineni et al., 2016). This is consistent with our results. In our study, the activation and mitochondrial translocation of cofilin-1 induced by α-synuclein fibrils ultimately leads to an increase in caspase 3 and cell apoptosis. Moreover, cofilin-1 was found to bind mitochondrial outer membrane receptor Tom 20, which helps transport proteins into mitochondria. The combination of cofilin-1 and Tom 20 is a key event in α-synuclein-mediated neuronal death. However, the question of whether the entry of cofilin-1 into mitochondria is mediated by Tom 20 remains speculative and requires further verification.

In addition, cofilin functions are also modulated by reactive oxygen species. Studies have shown that ROS-induced cofilin oxidation can induce oxidative stress in ischemic and hemorrhagic strokes (Alhadidi et al., 2016), and the oxidative stress-induced increase in cofilin dephosphorylation is linked to the accumulation of tau tangles and amyloid beta plaques in Alzheimer's disease (Namme et al., 2021). In our study, cofilin-1 also induces reactive oxygen species and their metabolites in PD cell models. Representative images depict the changes in oxidative stress. Mitochondrial damage generally aggravates the process of oxidative stress. The accumulation of ROS can lead to a variety of cellular stress reactions, including DNA damage and the corresponding apoptosis-related signal pathway activation (Hoffmann et al., 2021). It is foreseeable that excessive ROS production induced by cofilin-1 may become involved in aggravating cofilin-1 activation, thereby forming a vicious circle.

Overall, these results strongly suggest that α-synuclein inclusions promote the activation and overstimulation of cofilin-1 in mitochondria, which initiates a series of cytotoxic events, including the production of reactive oxygen species and mitochondrial membrane permeabilization. This leads to the release of apoptogenic factors from the mitochondria, including cytochrome c and apoptosis-inducing factor, which causes caspase 3 activation and triggers neuronal death. Our study describes the molecular mechanism of α-synuclein-mediated neuronal toxicity in mitochondria and provides an overview of cofilin's importance in PD physiology and pathophysiology. Molecular pathways that govern the neurotoxicity of α-synuclein aggregates are attractive therapeutic targets for PD. Phospho-regulation through SSHs and LIMKs is the most critical mechanism, where the Ser-3 residue of cofilin is the specific target. This novel biochemical pathway may thus be a good target for developing future therapeutic molecules for neurodegenerative diseases.

## Data availability statement

The original contributions presented in the study are included in the article/Supplementary material, further inquiries can be directed to the corresponding authors.

## Ethics statement

The animal study was approved by the Animal Care and Use Committee of Renmin Hospital of Wuhan University. The study was conducted in accordance with the local legislation and institutional requirements.

## Author contributions

MY: Writing – original draft. QZ: Writing – review & editing, Investigation. YC: Writing – review & editing, Investigation. CZ: Writing – review & editing, Methodology. DW: Writing – review & editing, Methodology. JT: Writing – review & editing, Data curation. BH: Writing – review & editing, Validation. QL: Investigation, Writing – review & editing. XD: Writing – review & editing. YW: Writing – review & editing.
